# Identification and Validation of Quantitative Trait Loci for *Wheat Dwarf Virus* Resistance in Wheat (*Triticum* spp.)

**DOI:** 10.3389/fpls.2022.828639

**Published:** 2022-04-14

**Authors:** Anne-Kathrin Pfrieme, Britta Ruckwied, Antje Habekuß, Torsten Will, Andreas Stahl, Klaus Pillen, Frank Ordon

**Affiliations:** ^1^Julius Kühn Institute (JKI) – Federal Research Centre for Cultivated Plants, Institute for Resistance Research and Stress Tolerance, Quedlinburg, Germany; ^2^Institute for Agricultural and Nutritional Sciences, Plant Breeding, Martin-Luther-University of Halle-Wittenberg, Halle (Saale), Germany; ^3^Julius Kühn Institute (JKI) – Federal Research Centre for Cultivated Plants, Quedlinburg, Germany

**Keywords:** *Wheat dwarf virus* (WDV), genome-wide association study (GWAS), wheat (*Triticum aestivum*), quantitative trait loci (QTL), resistance breeding, *Psammotettix alienus*

## Abstract

*Wheat dwarf virus* (WDV) is transmitted by the leafhopper *Psammotettix alienus.* As a major pathogen in wheat and other cereals, WDV causes high yield losses in many European countries. Due to climate change, insect-transmitted viruses will become more important and the restrictions in the use of insecticides efficient against *P. alienus* renders growing of WDV resistant/tolerant varieties the only effective strategy to control WDV. So far, there is little information about the possible sources of resistance and no known information about the genome regions responsible for the resistance. In a screening for WDV resistance using artificial inoculation in gauze houses, a panel of 500 wheat accessions including cultivars, gene bank accessions, and wild relatives of wheat was phenotyped for virus titer, infection rate, as well as plant height and yield parameters relative to healthy controls of the same genotype. Additionally, 85 *T. aestivum-Ae. tauschii* intogression lines were tested for WDV resistance in the greenhouse. A subset of 250 hexaploid wheat accessions was genotyped with the 15k iSelect SNP Chip. By genome-wide association study (GWAS), the quantitative trait loci (QTL) for partial WDV resistance were identified. Within these studies, one cultivar was identified showing an average infection rate of only 5.7%. By analyzing single seed descent (SSD) and doubled haploid (DH) populations comprising 153 and 314 individuals for WDV resistance and by genotyping these with the 25k iSelect SNP Chip, QTL for yield per plant, thousand-grain weight, and relative virus titer were validated on chromosomes 1B, 2B, 3B, 4B, 4A, 5A, 6A, and 7A. These results will be the basis for marker-assisted selection for WDV resistance to replacing the laborious, time-consuming, and technically challenging phenotyping with WDV bearing leafhoppers.

## Introduction

The phloem-limited *Wheat dwarf virus* (WDV) belongs to the genus of mastreviruses, family *Geminiviridae* (Fauquet et al., 2000), and was first described by Vacke in 1961 in former Czechoslovakia (Vacke, 1961; Lindsten and Vacke, 1991). It later became an important pathogen in other European countries as well as in Africa and Asia (Lindsten et al., 1970; Lapierre et al., 1991; Huth, 1994; Najar et al., 2000; Erlund, 2007; Xie et al., 2007; Ramsell et al., 2008; Wu et al., 2008; Liu et al., 2012). WDV is transmitted by the leafhopper *Psammotettix alienus* (Harrison et al., 1977). Host plants include members of the family *Poaceae*, such as various wild grasses and economically important cereals (Vacke, 1972).

Primary infection is caused by adult leafhoppers (*P. alienus*) in autumn and secondary infection in spring by nymphs (Lindblad and Sigvald, 2004). Longer warm periods in late summer and autumn promote infection pressure so that the early developmental stages of winter wheat and winter barley are particularly at risk (Mehner et al., 2003; Manurung et al., 2004). Symptoms of WDV infection in wheat include streaking of leaves, chlorosis, reduced number of ears, reduced winter hardiness, and death of plants at an early development stage (Vacke, 1972; Lindblad and Waern, 2002). The extent of damage depends strongly on the developmental stage at the time of infection. Infection at the two- to three-leaf stage leads to more pronounced symptoms with negative effects on winter hardiness and yield, while infections after shooting only lead to slightly shortened shoots (Lindblad and Waern, 2002).

The incidence of the disease in winter cereals varies from year to year. Symptomatic plants appear in patches on the field with mean yield losses of up to 35%. In some cases, local epidemics occur with yield losses of up to 90% (Fohrer et al., 1992; Lindsten and Lindsten, 1999; Lindblad and Waern, 2002; Širlová et al., 2005).

Due to a lack of resistant cultivars, only preventive agrotechnical measures, such as late sowing of winter cereals and reduction of the virus reservoir by removal of plant remains by plowing directly after harvesting, are available to control the spread of WDV in the field (Lindblad and Waern, 2002; Manurung et al., 2005). There is currently no approved insecticide available in the European Union for combating *P. alienus* and the pesticide application is expected to be further restricted, so the cultivation of tolerant/resistant varieties may be an evident and environment-friendly alternative in the future.

For this reason, resistance tests were carried out. Most of the tested winter wheat varieties were susceptible (Vacke and Cibulka, 2000). The least susceptible group was associated with an 82.5% yield reduction. In 2005, 25 registered winter wheat cultivars were tested for resistance in small plot tests. Here, the least susceptible varieties showed severe symptoms and heavy yield losses (87–93%) (Širlová et al., 2005). However, weak symptom expression with only mild chlorosis was observed in the Hungarian cultivars “Mv Dalma” and “Mv Vekni” (Benkovics et al., 2010). The virus titer of the infected plants determined by real-time PCR was lower in both genotypes than in the susceptible reference genotypes. Respective genotypes were classified as partially resistant due to their resistant characteristics under glasshouse conditions, but infectible under strong virus infection pressure in the field. Both genotypes also showed resistant properties to agro-inoculation with WDV DNA and no differences in insect survival or behavior compared to susceptible genotypes were observed. They represent the first identified sources of WDV resistance in wheat (Benkovics et al., 2010).

Due to the limited knowledge about WDV resistant wheat genotypes, combined genotyping and phenotyping accompanied by a subsequent association analysis will allow the identification of potential quantitative trait loci (QTL) for resistance. For example, the Wheat Infinium iSelect Beadchip offers thousands of single nucleotide polymorphism markers (SNPs) for genotyping (Wang et al., 2014). In wheat and other cereals, genome-wide association study (GWAS) has already been successfully applied to detect QTL for virus resistance, such as *Soil-borne wheat mosaic virus* (SBWMV) resistance in wheat (Liu et al., 2014), *Barley yellow dwarf virus* (BYDV) resistance in maize (Horn et al., 2014), BYDV resistance in oat (Foresman et al., 2016) and resistance to *Wheat spindle streak mosaic virus* (WSSMV) in wheat (Hourcade et al., 2019). Therefore, the aims of this study were to (i) screen a diverse collection of wheat genotypes for resistance to WDV, (ii) identify QTL for WDV resistance using genome-wide association studies, (iii) and verify these QTL in four biparental populations.

## Materials and Methods

### Leafhopper Rearing

The virus isolate “WDV-UA” which was isolated from a WDV-positive tested plant from Ukraine in 2010 (A. Habekuß, pers. commun., November 30, 2021) was used for the resistance screening, and the leafhopper species *P. alienus* served as WDV vector. For the inoculation of the biparental populations, leafhoppers were collected from the field (51°30′21.3 ″N 11°45′39.1 ″E) in 2018.

The virus vectors were kept in the greenhouse in perspex cages under controlled conditions (25°C, 50% relative humidity, 16 h photoperiod, 9,000 lux) and acquired the virus by feeding on WDV-infected wheat plants of the highly susceptible cultivars “Alcedo” and “Hybnos.” The WDV infection of these virus sources was regularly checked by using Double Antibody Sandwich-ELISA (DAS-ELISA). A transfer of the leafhoppers onto newly infected wheat plants was carried out every 6 weeks. For WDV inoculation of test plants, the leafhoppers were collected using a custom-made exhauster (Beco GmbH, Arnsberg, Germany).

### Plant Material

The gauze house screening panel of 500 wheat accessions (Supplementary Table S1) deriving from 59 countries consists of 363 wild and domesticated wheat species, mainly landraces and gene bank accessions [Leibniz Institute of Plant Genetics and Crop Plant Research (IPK), Gatersleben, Germany and National Small Grains Collection (NSGC), Aberdeen, WA, United States], 29 synthetic wheats derived from crosses of European *Triticum durum* lines and *Aegilops tauschii* [National Institute of Agricultural Botany (NIAB), Cambridge, United Kingdom] and 108 winter wheat varieties (Benkovics et al., 2010; Neumann et al., 2011). The gene bank accessions comprise the diploid species *Triticum boeoticum*, *T. urartu* and *T. monococcum*, tetraploid wheat such as *T. dicoccoides*, *T. araraticum*, *T. dicoccum, T. durum*, and *T. turgidum* as well as the hexaploid wheat *T. spelta, T. macha, T. vavilovii, T. sphaerococcum*, and *T. aestivum*. In addition, accessions of *Ae. bicornis, Ae. biuncialis, Ae. geniculata, Ae. kotschyi, Ae. longissima, Ae. peregrina, Ae. searsii, Ae. sharonensis*, and *Ae. triuncialis* species from Israel were included. 85 *T. aestivum—Ae. tauschii* introgression lines, containing different chromosome segments of *Ae. tauschii* (Pestsova et al., 2006), were tested in greenhouse inoculation tests.

Based on the results obtained in the screening (see below), the cultivar “Fisht” was crossed with susceptible varieties. “Fisht” is a released Russian wheat cultivar in the North-Caucasian region (S. Martynov, pers. commun., February 14, 2018). Single seed descent (SSD) and doubled haploid (DH) lines were developed by the plant breeding companies rouergue auvergne gévaudan tarnais (RAGT) and Strube Research. Verification of detected QTL was performed in four of the crosses. A total of 314 doubled-haploid lines, including 126 genotypes of the cross “Fisht” × “Faustus” (FixFa), 188 of the cross “Fisht” × “breedersline” (FixS), and a total of 153 SSD lines, including 78 of the cross “RGT Reform” × “Fisht” (FixRe), and 75 SSD lines of the backcross RexFi were tested.

### Experimental Design

#### Semi-Field and Greenhouse Experiments

The screening was based on the inoculation with virus-bearing leafhoppers and subsequent phenotyping for disease symptoms according to Vacke and Cibulka (2000). The resistance screening under semi-field conditions was conducted in gauze houses (L 30 m, W 6 m, H 3 m; with a pore size of 0.39 mm × 0.88 mm, Ornata Plus 3988, Howitec, Bolsward, Netherlands) in Quedlinburg, Germany (51°46′20.7′′N 11°08′46.5′′E). In total, 260 accessions were screened in 2014/2015 and another 240 accessions were tested in 2015/2016. In mid-September, the genotypes were sown in a randomized design with a row spacing of 20 cm in two variants per gauze house—one non-inoculated, healthy control variant, (15 seeds per accession) and one inoculated (WDV-infected) variant (15 seeds per accession) on which the viruliferous leafhoppers were released. The Hungarian winter wheat cultivars “Mv Emese,” “Mv Regiment,” “Mv Dalma,” and “Mv Vekni” were already characterized in regard to WDV inoculation (Benkovics et al., 2010) and therefore included in each screening test as susceptible and partially resistant standard varieties, respectively. These standards and the variety “Fisht” which showed low WDV infection rates in a pre-screening, were sown between the tested accessions at the beginning, in the middle, and at the end of each trial. Before the inoculation took place, wheat plants carrying WDV were planted between the rows as additional virus sources for the leafhopper vectors in order to optimize the injection pressure.

When the two- to the three-leaf stage was reached in October, both variants were covered by a fleece (Type 17, 18 g/m^2^, Schachtrupp KG, Schenefeld, Germany) and WDV transmitting leafhoppers were released under the cover of the inoculated variant with a colonization rate of about one insect per plant. After 6 weeks, the inoculated variant was uncovered first and sprayed with an insecticide (0.035% Confidor, Bayer Crop Science AG, Monheim, Germany) followed by the same treatment of the control variant in order to avoid unintended infection. If necessary, the plants were repeatedly treated with Confidor to avoid BYDV infections by aphids and with a fungicide (0.1% Corbel, BASF, Ludwigshafen, Germany) against powdery mildew (*Blumeria graminis*). Accessions with a tendency to lodge were fixed by bamboo sticks. Manual harvesting took place at the end of July.

Accessions showing a WDV infection rate ≤ 20% measured in November by DAS-ELISA and a weak visual symptom expression were selected for a replication test. When screening the second panel in 2015/2016, some spare capacity in the gauze houses was used to conduct a first single replication test with 40 selected accessions out of the first screening in 2014/2015. In the third season in 2016/2017 a replication test including the 38 best accessions according to the mentioned criteria out of all tests was conducted in a randomized block design with two replications per accession. Due to the weather conditions in the season 2016/2017, the inoculation was carried out in the greenhouse. At the two-leaf stage, each plant was covered with a perspex tube together with two viruliferous leafhoppers for 1 week at 15°C. After the removal of the vectors and spraying with an insecticide (0.1% Pirimor, Syngenta Agro, Maintal, Germany) and fungicide (0.1% Corbel, BASF, Ludwigshafen, Germany), six infected and six control plants of each accession were vernalized for 8 weeks at 4°C in a climate chamber and planted into the gauze house. In addition, the 85 introgression lines were inoculated as described but were grown in the greenhouse and tested by DAS-ELISA after 5 weeks when the first virus symptoms were visible.

For the verification of the QTL identified by GWAS, resistance tests were carried out in 2018/2019, 2019/2020, and 2020/2021 in gauze houses according to the procedure of Pfrieme et al. (in prep.). Per gauze house, 90 genotypes including standards were tested. Twelve plants each were arranged in a randomized block design with a row spacing of 20 cm in the inoculated (WDV-infected) and non-inoculated (non-infected control) variant. In the first year, inoculation was carried out under semi-field conditions in the gauze houses. For this purpose, twelve seeds per treatment and line were sown directly into the gauze house in October 2018 in Quedlinburg (51°46’20.7 ″N 11°08’46.5 ″E). At BBCH 10 (Meier, 2001), a separate tunnel system was set up in the gauze house for inoculation, covering plants for each treatment individually. To achieve this, steel arches were fixed in the ground and covered with double fleece. The infested variant was inoculated for 4 weeks during tillering (BBCH 10-23). The colonization rate corresponded to about one leafhopper per plant. After the first frost day, the infected treatment was uncovered again and the plants were treated with an insecticide (Pirimor G^®^, Adama, concentration 0.1%) and a fungicide (Flexity^®^, BASF, concentration 0.1%). One week later, the gauze tunnel of the control treatment was removed and the plants were treated as described.

In 2019/2020 and 2020/2021, inoculation took place in the greenhouse using an inoculation hood. For the trials, ten grains were used for each line and treatment. After a germination period of 3 days, the plants were covered with the hood and colonized with 30 leafhoppers, 15 adults, and 15 nymphs each. This corresponds to a population of 0.5 leafhoppers per plant. Inoculation took place over a period of 14 days after which the leafhoppers were removed. Plants of both variants were treated with the mentioned insecticide and fungicide. Subsequently, plants were planted in a gauze house by hand.

#### Assessment of *Wheat Dwarf Virus* Infection

The virus titer of each plant was measured in November (BBCH 23-30) and May (BBCH 51-55) by DAS-ELISA according to Clark and Adams (1977) using microplates coated with custom-made polyclonal antibodies (Julius Kühn-Institute, Quedlinburg/Germany). The analysis was performed with 50 mg plant material per sample. The extinction value (E) was measured photometrically at 405 nm 60 min after the addition of the enzyme-substrate (p-nitrophenylphosphate) with a microtiter plate reader (Thermolab Systems Opsys MR, Dynex Technologies, Chantilly, VA, United States for the screening, and Tecan Sunrise, Tecan, Männedorf, Switzerland for the analysis of the biparental populations). The extinction intensity is a measure of the relative virus titer. The leaf samples of healthy wheat plants (lyophilized leaf samples) were used as negative controls. Positive infections were defined using a calculated cut-off value for extinction (Equation 1, Lardeux et al., 2016) in the DAS-ELISA.


(1) $$\mathrm{Cut}-\mathrm{off}=\mathrm{mean}\,\bar{X}\,\left(\mathrm{mean}\,\mathrm{negative}\,\mathrm{control}\right)+3\,x\,\mathrm{standard}\,\mathrm{deviation}\,\mathrm{of}\,\mathrm{negative}\,\mathrm{control}$$

The infection rate in % (IR) was determined by the number of WDV infected plants divided by the total number of plants per accession in the infected variant and multiplied by 100. The mean of the extinction values of infected plants per accession was referred to as the mean relative titer. Plants with an extinction value below the cut-off were excluded from the calculation of the mean relative virus titer.

#### Visual Scoring of Infection and Measurement of Agronomic Parameters

**FIGURE 1:**
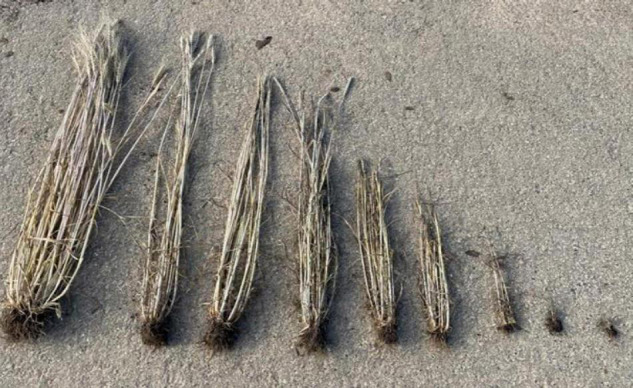
Symptom scoring, from left to right 1–9; 1-asymptomatic, 2-very weak growth reduction, normal tillering; 3-weak growth reduction, normal tillering, marked yellowing of the leaf tips (not shown); 4-weak growth reduction, reduced number of ears; 5-significant reduction in growth, reduced number of tillers and spike-bearing stalks; 6- weak growth reduction, greatly reduced number of shoots and ears; 7-plant with one to four shoots, no ears or ears stuck in the bract, strongly dwarfed; 8-plant without shoots, no spikes, heavily dwarfed; 9-plant dead.

Various agronomic parameters were recorded during the gauze house tests. Between tillering and shooting (BBCH 23-30), a comparative symptom assessment (Figure 1) between plants of the inoculated variant and the control was carried out and the extinction (E) was determined. The heading date of the infected and the control plants was recorded when 75% of the accessions reached BBCH 59 in order to determine the delay in heading due to WDV infection.

After harvest, the number of plants for each genotype was counted and plant height, the number of ears, and yield was determined separately for the infected and control variant. Plants with an extinction value below the cut-off were excluded from analysis because they were considered as uninfected (no accessions without any infected plants were observed after completion of all resistance tests). The thousand kernel weight (TKW) was calculated using the MARVIN seed analyzer (GTA Sensorik GmbH, Neubrandenburg, Germany). The relative parameters for plant height (relPH), number of ears per plant (relEars), grain yield per plant (relYield), and thousand kernel weight (relTKW) were calculated by comparing the mean performance of WDV infected plants for each accession to the mean performance of the healthy control (Equation 2). The number of grains per ear (relGrains/Ear) was calculated based on the number of ears, yield and TKW (Equation 3). In addition, the reaction to a WDV infection was assessed using a rating scale from 1 = healthy plant to 9 = plant died (Figure 1).


(2) $$\mathrm{Relative}\mathrm{performance}\left(\%\right)=\frac{\mathrm{Mean}\,\mathrm{of}\,\mathrm{WDV}\,\mathrm{infected}\,\mathrm{plants}}{\mathrm{Mean}\,\mathrm{of}\,\mathrm{control}\,\mathrm{plants}}\times 100\%$$

(3) $$\mathrm{Number}\,\mathrm{of}\,\mathrm{grains}\,\mathrm{per}\,\mathrm{ear}=\frac{\mathrm{Yield}\,\left(g\right)\times 1000}{\mathrm{TKW}\,\left(g\right)\times \mathrm{Number}\,\mathrm{of}\,\mathrm{ears}}$$

### Data Analysis

A two-way ANOVA with the variables “accession” and “year” as factors using the program Genstat (Genstat for Windows 19th Edition. VSN International, Hemel Hempstead, United Kingdom) was conducted for the phenotypic data. The ANOVA was calculated separately for accessions tested in 3 years (year 1 + 2 + 3) and in 2 years (year 2 + 3) including the standard cultivars. A Shapiro–Wilk test for normal distribution was conducted and additionally, diagnostic plots for normal distribution and variance homogeneity were checked. The 2-year data for the trait infection rate were analyzed without transformation and the 3-year data were square-root transformed before ANOVA in order to achieve a normal distribution of residuals. The traits relPH, relEars, relYield, relGrains/Ear, and relTKW were not analyzed by ANOVA because the required assumptions of variance homogeneity and normally distributed residuals were not obtained even after transformation of data (log-, logit-, square root-transformation). For these traits, many zero percent values were calculated for the relative performance of WDV infected accessions since these plants did not fully develop to maturity due to the virus infection.

The virus infection was correlated to the yield-related parameters and the plant height relative to the healthy controls by Pearson’s correlation coefficient test using R version 3.1.2, Vienna, Austria (R-Core-Team, 2014).

### Genome-Wide Association Study

The subpanel for the GWAS covers different degrees of susceptibility to WDV (IR = 3–100%) including 14 hexaploid accessions showing ≤ 20% infected plants. The phenotypic data of the overall-means for the traits IR, E, relPH, relEars, relYield, relGrains/Ear, and relTKW of each accession based on the resistance screening and (if available) the replicated tests were entered into the analysis (data online at OpenAgrar: https://doi.org/10.5073/20220203-155132). Genomic DNA was isolated according to a modified Cetyltrimethylammoniumbromid (CTAB) method (Doyle and Doyle, 1987). We used about 300 mg plant material from the youngest leaves which were macerated with 1.2 ml extraction buffer in a plastic bag (5 × 10 cm, Semadeni AG, Swiss). For genotyping by the 15k iSelect BeadChip containing 13,006 SNPs (SGS Institute Fresenius GmbH, Trait Genetics Section, Gatersleben, Germany), the samples were adjusted to a DNA concentration of 50 ng/μl. The data were filtered for polymorphic SNP markers which are mapped on the 90k consensus map (Wang et al., 2014) and show minor allele frequencies (MAF) > 3%, heterozygosity < 12.5%, and missing data < 10%. With the program Beagle version 3.2.2, Washington, United States (Browning and Browning, 2007), missing values > 10% were imputed. A principal component analysis (PCA) to detect population structure was performed using DARwin version 5.0, Montpellier, France (Perrier and Jacquemoud-Collet, 2006). The resulting 10,568 SNPs and a kinship matrix (K-matrix) based on Roger’s distance to determine the relatedness was calculated with the software package R version 3.1.2, Vienna, Austria (R-Core-Team, 2014). The GWAS was performed on the basis of the kinship matrix, the genotypic data of 10,568 SNPs, and the phenotypic data of the association panel by TASSEL 4.1 (Bradbury et al., 2007). For the identification of significant marker trait associations (MTAs), the compressed mixed linear model corrected for the K matrix (CMLM + K) was selected (Zhang et al., 2010). We set a first significance threshold of -log10 (*p*-value) = 3.0 and a more stringent threshold by the false discovery rate (FDR, α < 0.05) which corrects for multiple testing (Osborne, 2006; Zanke et al., 2015) for the detection of highly reliable MTAs. The linkage disequilibrium LD (r^2^) was determined R version 3.1.2, Vienna, Austria (R-Core-Team, 2014). For QTL identification, the most significantly associated markers with *p*-values above the FDR threshold were defined as QTL peak markers, and the phenotypic variance explained by markers (R^2^) and allelic effects were estimated by TASSEL 4.1 (Bradbury et al., 2007; Maccaferri et al., 2015).

### Gene Annotation

Since most of the markers used in this study have already been mapped, the chromosome to which they were assigned is known. Physical QTL intervals for gene annotation were set according to the expected LD decay of 1 Mb on either side of the peak marker, i.e., 2 Mb interval in total (Yu et al., 2020; Jan et al., 2021). Physical locations of peak markers were identified by anchoring these to the physical reference sequence IWGSC RefSeq v1.0 (Appels et al., 2018) and QTL intervals were searched for corresponding v1.0 annotations using WheatMine search (Alaux et al., 2018).

### Quantitative Trait Loci Validation

The QTL obtained in the GWAS were validated using two DH and two SSD populations resulting from crosses between “Fisht” and susceptible varieties (see above). These were grown without replication in gauze houses, manually infected, and subjected to validation. The traits relPH, relEars, relShoots, relYield, relTKW, and relative virus titer (E) were determined as described above. Genomic DNA was isolated from all tested genotypes using the method described above and samples were adjusted to a DNA concentration of 50 ng/μl. The Illumina 25k iSelect BeadChip (SGS Institute Fresenius GmbH, Trait Genetics Section, Gaersleben, Germany) containing 24,146 SNPs was used for genotyping. The filtering of the data was done as described for the association analysis but with a MAF >5%. To analyze the data, a Shapiro–Wilk-*W* test for normal distribution was performed, first. If a normal distribution was observed, a Levene test was performed to determine variance homogeneity.

For normally distributed traits, differences between the means of the phenotypic values of the two alleles were tested using a *t*-test for each combination of QTL and population. Non-normally distributed or inhomogeneous traits were analyzed with a Mann–Whitney test. A false discovery rate of 5% was assumed as the threshold for significance.

In addition, the effects of the selected markers for traits of special interest in breeding, relYield, relTKW, and E, were estimated using a multiple regression model. The regression models were built based on the four combined populations (Pop_total) or for each population separately (Pop_each). The models were used to predict the values of the individual lines. The coefficients of correlation between the predicted and the actual values were determined (R-Core-Team, 2020).

## Results

### Genotypic Variability in Response to a *Wheat Dwarf Virus* Infection

The semi-outdoor inoculation efficiency in the gauze houses reached an average infection rate of 72% for the susceptible variety “Hybnos”. In all tests, we observed increasing infection rates from November to May. In accordance with the study of Benkovics et al. (2010), the average proportion of infected plants was high for the varieties “Mv Emese” (68.1%) and “Mv Regiment” (64.9%) whereas “Mv Dalma” (34.5%) and “Mv Vekni” (21.5%) showed much lower infection rates. A broad distribution of infection rates ranging from 3 to 100% in domesticated wheat accessions and from 0 to 100% in wild relatives was identified after gauze house tests but the majority showed high susceptibility in the range of the susceptible standard varieties. A total of 264 wheat accessions died completely until July after being infected with WDV. In all remaining infected accessions, the virus caused a delay in the heading date varying from 2 to 27 days. In the two-way ANOVA, a significant effect (*p* < 0.001) of “accession” and “year” on the infection rate was detected for the selected accessions tested in 2 and 3 years (Table 1). The interaction “accession x year” was not significant.

**TABLE 1:** Two-way ANOVA results of selected accessions including the varieties “Hybnos,” “MV Emese,” “MV Regiment,” “Mv Dalma,” and “Mv Vekni” tested in 2 years and 3 years, respectively, showing the effect of accession, year and interaction on the WDV infection rate.

ANOVA	Source	*F* value	*P* value
Test statistics for 3-year data	Accession	8.03	<0.001***
	Year	16.50	<0.001***
	Accession × Year	1.36	0.075
Test statistics for 2-year data	Accession	5.79	<0.001***
	Year	26.65	<0.001***
	Accession × Year	1.96	0.052

*p < 0.05, **p < 0.01, and ***p < 0.001.

**FIGURE 2:**
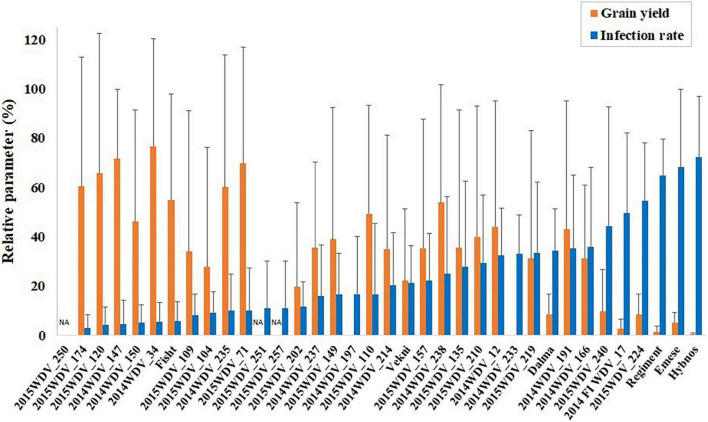
Mean of infection rate (%) in May and relative grain yield (%) of the accessions tested in project year 1 + 2 + 3 or in project year 2 + 3, respectively, in comparison to the partially resistant varieties “Mv Dalma” and “Mv Vekni” and the susceptible varieties “Mv Regiment,” “Mv Emese,” and “Hybnos.” NA, yield not measurable due to spindle brittleness.

Out of the 36 accessions tested in the ANOVA (Supplementary Table S2), 19 accessions of different species including ten *T. aestivum*, two *T. vavilovii*, two *T*. sp. (gene bank accessions with unknown subspecies), one *T. boeoticum*, one *T. macha*, one *Ae. geniculata*, one *Ae. bicornis*, and one *Ae. longissima* accession were identified with a lower infection rate than the partially resistant “Mv Vekni” (21.5%). Despite relatively large residuals, the hexaploid accessions TRI 4630 (2014WDV_147, *T. vavilovii*, China), TRI 9632 (2014WDV_150, *T. vavilovii*, Former Soviet Union), PI 245511 (2014WDV_34, *T. aestivum*, Afghanistan), and the winter wheat cultivar “Fisht” had a low infection rate of 4.4–5.7% and a relYield of 46.2–76.8% over three seasons of testing (Figure 2).

Two Israeli *Aegilops* species tested in multiple years (*Ae. longissima*, 2015WDV_251 and *Ae. bicornis*, 2015WDV_257) showed a relatively low mean infection rate of 11%. Their relative yield is not shown due to spindle brittleness. The accession 2015WDV_250 (*Ae. geniculata*, Israel) was not infected in the gauze house tests and no symptoms were scored (score = 1.0), however, in the greenhouse, this accession showed some infection (IR = 16.7%). The *T. aestivum*-*Ae. tauschii* introgression lines tested in the greenhouse showed high mean infection rates (33–100%), severe visible symptom expression, and high average extinction values (*E* = 0.8–1.6).

**FIGURE 3:**
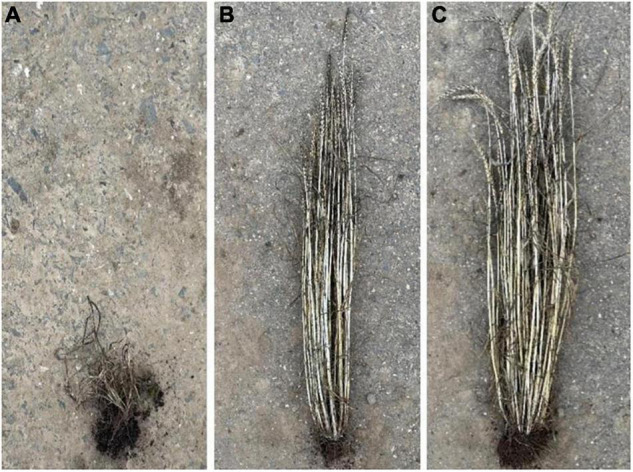
Visual comparison of an infected “Hybnos” plant **(A)** after harvest of the gauze house test and two infected “Fisht” plants with a wheat dwarf virus (WDV) extinction value of *E* = 0.31 **(B)** and *E* = 0.03 **(C)**, respectively.

In contrast, the low average number of infected plants (5.7%) in the released Russian winter wheat cultivar “Fisht” was accompanied by less severe virus symptoms in May (average score 2.3) compared to the partially resistant varieties “Mv Dalma” (5.9) and “Mv Vekni” (4.6), and the susceptible standards “Mv Regiment” (6.7), “Mv Emese” (6.9), and “Hybnos” (7.9). The pictures (Figures 3 A–C) show the infected plants of “Fisht” which differ in the symptom expression in dependence of the virus titer but had much weaker virus symptoms compared to the infected “Hybnos.”

### Genome-Wide Association Study for *Wheat Dwarf Virus* Resistance of 250 Hexaploid *Triticum* Accessions

The GWAS panel consisted primarily of *T. aestivum* and some accessions of *T. macha*, *T. spelta*, *T. vavilovii*, and *T. sphaerococcum* which were medium to highly susceptible, and only 6% less susceptible accessions identified with an IR less than “Mv Vekni” (21.5%). The virus extinction and infection rates were strongly negatively correlated with the plant height and all yield-related parameters (Table 2).

**TABLE 2:** Summary of the Pearson correlation matrix for extinction (E), infection rate (IR), relative plant height (relPH), relative number of ears per plant (relEars), relative number of grains per ear (relGrains/Ear), relative grain yield (relYield), and relative thousand kernel weight (relTKW) of 250 wheat accessions tested for WDV resistance.

Trait	E	IR	relPH	relEars	relGrains/Ear	relYield	relTKW
E	1						
IR	0.43	1					
PH	−0.55	−0.62	1				
Ears	−0.52	−0.50	0.87	1			
Grains/Ear	−0.49	−0.46	0.77	0.72	1		
Yield	−0.66	−0.59	0.81	0.78	0.79	1	
TKW	−0.40	−0.42	0.80	0.79	0.78	0.63	1

*All correlations are significant to p < 0.01.*

**FIGURE 4:**
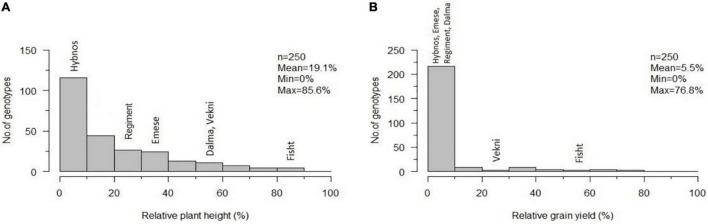
Frequency distribution of accession means for relative plant height **(A)** and relative grain yield per plant **(B)** after artificial WDV infection of 250 hexaploid wheat accessions in comparison to the susceptible standard cultivars “Hybnos,” “Mv Regiment,” and “Mv Emese” and the partially resistant cultivars “Mv Dalma” and “Mv Vekni”. The variety “Fisht” is highlighted separately. n, number of tested accessions; Mean; Min; Max, overall mean, minimum, and maximum of the trait relative plant height **(A)** and relative grain yield **(B)**.

The distribution of relative plant height and relative yield shows a strong effect of WDV on most accessions (Figures 4 A,B). The partially resistant cvs. “Mv Dalma” and “Mv Vekni” showed 8.7% and 22.1% relYield, respectively. In comparison, “Fisht” had a higher relative grain yield under WDV infection (relYield = 54.9%) as well as higher relEars = 78.6%, relTKW = 82.7%, and relGrains/Ear = 65.3%. In addition, five accessions, namely, PI 245511 (*T. aestivum*, Afghanistan), TRI 4630 (*T. vavilovii*, China), Qinfeng 208 (*T. aestivum*, China), Inia 66 (*T. aestivum*, Mexico), and Vel (*T. aestivum*, United States) were identified, showing only 3–10% infected plants along with 55–77% relYield.

**FIGURE 5:**
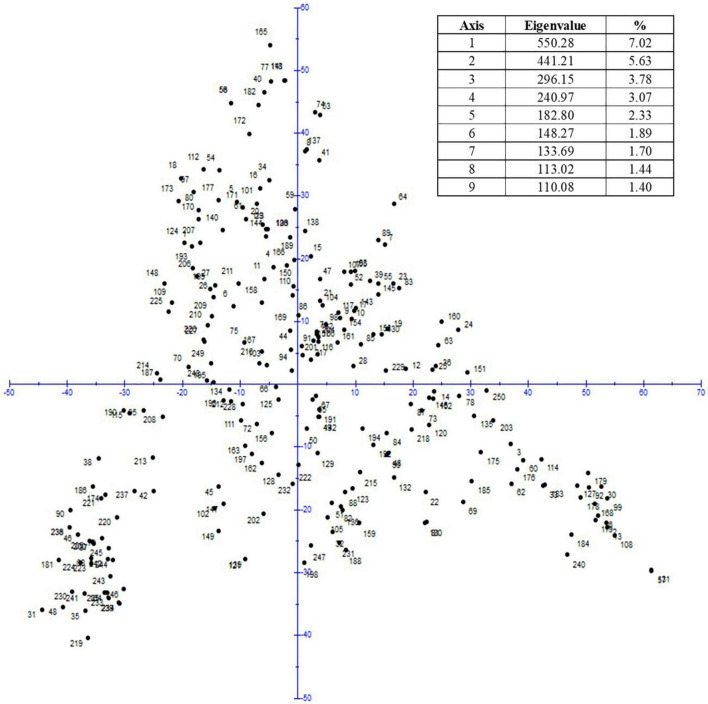
Principal component analysis.

For the GWAS, 10,568 polymorphic SNP markers were used whereby the marker density on the D genome was low resulting in an under-representation of these chromosomes (Supplementary Table S3). The LD decay was determined to be 2.97 cM (Supplementary Figure S1). The PCA revealed no strong population structure (Figure 5) and thus, only the K matrix was considered for GWAS.

For all investigated traits, in summary, 244 significant MTAs (*p* < 0.001) were detected for resistance to WDV and 47 MTAs after FDR correction (α < 0.05) corresponding to 35 peak markers were identified for relPH, relYield, and relTKW, as illustrated by the Manhattan plots (Supplementary Figure S2). For the trait relPH, 26 QTLs were detected on chromosomes 1B, 1D, 2B, 3A, 3B, 4A, 4B, 5A, 6A, 7A, and 7B, seven QTL for relYield on chromosomes 1B, 2B, and 3A and two QTL for relTKW on chromosome 5A (Table 3). The phenotypic variance explained by a single marker ranged from 7.0 to 18.3%. Up to six additional significantly associated markers (LOD > 3) were found within the QTL intervals. Four QTL for the traits relPH and relYield located on chromosomes 3A and 1B share the same position and significant SNP markers (Table 3). The most significant QTL were identified on chromosome 1B (WDV_Yield_1B1 and WDV_Yield_1B3) with a LOD of 8.7 and the highest phenotypic variance explained (18.3%).

**TABLE 3:** Quantitative trait loci (QTL) and corresponding significant marker-trait associations [false discovery rate (FDR), α < 0.05] for resistance to WDV in hexaploid wheat accessions detected for the relative traits plant height (WDV_PH), grain yield (WDV_Yield), and thousand kernel weight (WDV_TKW).

QTL	Peak marker	SNP	Chr.	Pos. (cM)	QTL interval (cM)	MAF	LOD	R^2^ (%)	PIC	Allelic effect
**WDV_PH_1B1**	Ku_c31363_2165	A/**C**	1B	31.0	29.6–32.5	0.198	4.4	8.8	0.267	19.0
**WDV_PH_1B2**	**Excalibur_c12376_ 569**	C/**T**	1B	43.9	42.4–45.3	0.106	4.6	9.4	0.172	28.1
**WDV_PH_1B3**	**wsnp_Ex_c2117_3976893**	G/**T**	1B	53.5	52.0–55.0	0.204	4.2	7.0	0.272	13.8
**WDV_PH_1B4**	**Kukri_c26168_423**	**A**/C	1B	60.6	59.1–62.1	0.106	4.6	9.4	0.172	28.1
**WDV_PH_1B5**	BS00068182_51	**A**/G	1B	62.6	61.1–64.1	0.188	3.9	7.8	0.259	21.1
WDV_PH_1B6	Tdurum_contig60509_ 232	C/**T**	1B	74.4	72.9–75.9	0.436	4.0	8.1	0.371	5.5
WDV_PH_1D1	Ra_c11906_1441	**A**/G	1D	107.1	105.6–108.5	0.174	4.4	8.9	0.246	31.1
**WDV_PH_2B1**	BS00009882_51	**A**/G	2B	134.5	133.0–135.9	0.256	4.1	8.3	0.308	14.6
**WDV_PH_3A1**	**Excalibur_c48047_90**	**A**/G	3A	101.0	99.5–102.5	0.246	5.8	11.9	0.302	20.2
**WDV_PH_3B1**	Excalibur_c9001_569	A/**G**	3B	65.7	64.2–67.2	0.384	4.4	8.9	0.361	26.4
**WDV_PH_3B2**	wsnp_Ex_c2609_4852360	**C**/T	3B	73.3	71.8–74.7	0.300	4.7	9.5	0.332	14.1
**WDV_PH_3B3**	IAAV3519	**A**/G	3B	79.9	78.5–81.4	0.238	4.2	8.5	0.297	24.2
WDV_PH_3B4	BobWhite_c24364_73	G/**T**	3B	85.5	84.0–87.0	0.174	4.5	9.2	0.246	23.6
WDV_PH_4A1	RAC875_rep_c117027_ 577	**G**/T	4A	147.2	145.7–148.6	0.412	3.9	8.0	0.367	2.0
WDV_PH_4A2	Excalibur_rep_c69170_ 425	G/**T**	4A	151.2	149.7–152.6	0.344	4.8	9.8	0.349	10.3
**WDV_PH_4A3**	BobWhite_c25163_178	A/**G**	4A	153.0	151.5–154.5	0.460	5.0	10.3	0.373	11.9
**WDV_PH_4B1**	wsnp_BE638137B_Ta_ 2_2	**C**/T	4B	114.9	113.4–116.4	0.328	3.8	7.7	0.344	15.7
**WDV_PH_5A1**	Kukri_c3338_271	**C**/T	5A	46.1	44.6–47.5	0.416	4.2	8.5	0.368	16.4
**WDV_PH_5A2**	BS00076190_51	**A**/G	5A	50.6	49.1–52.1	0.346	4.0	8.1	0.350	11.8
**WDV_PH_5A3**	Excalibur_c8030_2139	**G**/T	5A	84.1	82.6–85.6	0.492	4.1	8.2	0.375	6.2
WDV_PH_5A4	RAC875_c26353_719	A/**G**	5A	91.7	90.2–93.2	0.334	5.7	11.6	0.346	13.3
**WDV_PH_6A1**	RAC875_rep_c105861_ 454	**C**/T	6A	136.7	135.2–138.1	0.236	4.2	8.5	0.296	16.2
**WDV_PH_7A1**	Tdurum_contig49186_ 437	A/**G**	7A	65.3	63.8–66.8	0.314	4.4	8.8	0.338	9.2
**WDV_PH_7A2**	Excalibur_rep_c68955_ 286	**A**/G	7A	74.2	72.7–75.7	0.152	4.1	8.4	0.225	32.4
**WDV_PH_7A3**	wsnp_Ex_c5839_ 10246915	**C**/T	7A	212.7	211.2–214.1	0.496	3.9	7.8	0.375	11.8
WDV_PH_7B1	RAC875_c906_657	**C**/T	7B	167.6	166.1–169.0	0.408	4.7	8.1	0.366	11.7
**WDV_Yield_1B1**	**Excalibur_c12376_569**	C/**T**	1B	43.9	42.4–45.3	0.106	8.7	18.3	0.172	22.3
**WDV_Yield_1B2**	**wsnp_Ex_c2117_3976893**	G/**T**	1B	53.5	52.0–55.0	0.204	4.9	8.3	0.272	10.5
**WDV_Yield_1B3**	**Kukri_c26168_423**	**A**/C	1B	60.6	59.1–62.1	0.106	8.7	18.3	0.172	22.3
**WDV_Yield_1B4**	Tdurum_contig44861_ 1253	A/**C**	1B	62.6	61.1–64.1	0.176	4.4	7.4	0.248	10.1
**WDV_Yield_2B1**	BS00071690_51	**A**/G	2B	82.0	80.5–83.4	0.158	5.0	10.1	0.231	20.0
**WDV_Yield_2B2**	RAC875_rep_c83950_ 222	C/**T**	2B	144.1	142.7–145.6	0.410	7.6	15.9	0.367	27.5
WDV_Yield_3A1	**Excalibur_c48047_90**	**A**/G	3A	101.0	99.5–102.5	0.246	6.0	15.9	0.302	12.6
WDV_TKW_5A1	BS00094342_51	G/**T**	5A	60.8	59.3–62.3	0.312	5.4	11.2	0.337	17.9
WDV_TKW_5A2	IAAV5294	C/**T**	5A	62.7	61.2–64.2	0.222	4.5	9.3	0.286	1.8

*Chr., chromosome; Pos., position in cM; QTL interval according to LD = 2.972 (+/−1.486 cM); MAF, minor allele frequency of each marker; LOD, −log10 (P-value); R^2^, phenotypic variance explained by marker in percentage; PIC, polymorphism information content.*

*QTLs highlighted in bold have been validated by analysis of biparental populations.*

*Peak markers highlighted in bold have been found in QTL for both traits, WDV_PH and WDV_Yield.*

*In the column “SNP,” the favorable allele is marked in bold.*

### Quantitative Trait Loci Annotation

Locations on the physical wheat map were identified for 27 putative QTL regions. By annotating the QTL peak markers ± 1 Mb, genes were identified, which have already been associated with various stress responses in plants. For all QTLs, the associated peak markers are located within high confidence genes based on the wheat genome reference sequence IWGSC RefSeq v1.0 (Supplementary Table S4). The majority of the identified genomic regions are located on chromosome 1B. In this respect, genes encoding DNA template regulation of transcription (WDV_PH_5A4, WDV_Yield_1B3, WDV_TKW_5A1), gene silencing by RNA (WDV_PH_5A4), and protein kinase activity (WDV_PH_5A4, WDV_Yield_1B3, WDV_Yield_2B1, WDV_Yield_2B2) were found to be associated with those QTL explaining > 10% phenotypic variance.

### Validation of Quantitative Trait Loci

To validate the QTL discovered by association mapping, we performed linkage analysis with four biparental populations (FixFa, FixS, FixRe, RexFi; Supplementary Table S5A). “Fisht” was selected as a crossing partner because it is the only wheat cultivar showing resistance to WDV identified within the screening panel which might be easier integrated into resistance breeding compared to wild gene bank accessions. Only DH/SSD lines with at least three infected plants were included in the evaluation. This resulted in 56 genotypes of the cross FixFa, 59 of the cross FixRe, 23 of the cross RexFi, and 53 of the cross FixS. The distribution of the traits relYield, relTKW and E in the populations is shown in Supplementary Figure S3.

The correlation values of the individual traits clearly show the influence of the WDV infection on the individual yield-determining traits. The relative virus titer correlates negatively with most phenotypic traits (Supplementary Table S6).

For QTL validation, we used the genotyping data already available for these populations based on the 25k iSelect chip. Validation was carried out for 25 of the 35 identified QTL (Table 3, marked in bold). In 19 QTL the favorable alleles derived from the resistant donor parent “Fisht” (Supplementary Table S5B).

Two QTL for relYield and ten for the extinction value (relative virus titer) showed significant effects to varying degrees across all populations. The most significant effects were observed for TKW with 11 significant QTL (Table 4 and Supplementary Table S7). Looking at significant differences at the population level, most were observed for the FixFa population. In particular, one QTL for relYield on chromosome 1B (WDV_Yield_1B1) showed highly significant and consistent effects in all populations. The contributions of WDV_PH_1B1-1B5 and WDV_Yield_1B1-WDV_Yield_1B4 were significant in three populations. The effects of WDV_PH_7A1 were only validated in two populations. An effect of WDV_PH_3B1 and WDV_PH_6A1 was only confirmed in the FixFa population.

**TABLE 4:** Validations of the identified QTL using a *t*-Test, *, ** indicate significant differences between allele means at 5% and 1% level, respectively.

	Population total			Population each											
	total			FixFa			FixS			FixRe			RexFi		
	P			P			P			P			P		
QTL	refYield	relTKW	E	refYield	relTKW	E	relYield	relTKW	E	relYield	relTKW	E	refYield	relTKW	E
WDV_PH_1B1	0,015*		0,035	0,003*	0,003*							0,035			
WDV_PH_1B2	0,021		0,023*	0.006*	0,004							0,023			
WDV_PH_1B3	0,017*		0,027*	0,003*	0,003*							0,027*			
WDV_PH_1B4	0,021		0,035*	0,003*	0,003*							0,035			
WDV_PH_1B5	0,031			0,003*	0,003*							0.03''			
WDV_PH_2B1	0,008**														
WDV_PH_3A1															
WDV_PH_3B1	0,001**	0,015*				0,01''									
WDV_PH_3B2															
WDV_PH_3B3															
WDV_PH_4A3			0,006**												
WDV_PH_4B1															
WDV_PH_5A1															
WDV_PH_5A2															
WDV_PH_5A3															
WDV_PH_6A1			0,0001**			0,032*									
WDV_PH_7A1	0,02*														
WDV_PH_7A2															
WDV_PH_7A3															
WDV_Yield_1B1	0,021		0,023	0,01	0,008**							0,036			
WDV_Yield_1B2	0,017		0,027*	0,01	0,009**							0,031			
WDV_Yield_1B3	0,021		0,035	0,003**	0,007**							0,036			
WDV_Yield_1B4	0,026		0,027	0,01	0,008**	0,03''									
WDV_Yield_2B1															
WDV_Yield_2B2															

The multiple regression analysis, using the Pop_total data set based on the combination of all populations (Supplementary Table S8) revealed significant effects for one QTL for relYield, one QTL for relTKW, and three QTL for E. Within the population FixFa, three QTL for relYield, one QTL for relTKW, and two for E turned out to be significant. Using the FixS dataset, one QTL for relYield was significant.

The regression models were used to predict the traits relYield, relTKW and E in the individual genotypes (Supplementary Table S9). The coefficients of determination were 0.2122; 0.4476; 0.3233 for the trait relYield in the Pop_total, FixFa, FixS populations, 0.2121; 0.3943; 0.1894 for the trait relTKW in the Pop_total, FixFa, and FixS populations and 0.2671; 0.4343; 0.2818; 0.7392 for the trait E in the Pop_total, FixFa, FixS, and FixRe populations.

In each population, the prediction accuracy of the traits relYield and relTKW was higher based on the FixFa dataset than on the Pop_total dataset. The highest prediction accuracy was obtained for E (Supplementary Figure S4).

## Discussion

### Variability in Response to *Wheat Dwarf Virus* Infection

The aim of this study was to identify wheat genotypes with a high level of resistance to WDV and to get information on the genetic basis of resistance to WDV. The majority of previous studies reported high susceptibility to WDV in registered wheat varieties ranging from 87 to 100% yield losses when infected in the tillering stage (Vacke and Cibulka, 2000; Širlová et al., 2005). Analogous to wheat, barley varieties showed a high yield reduction (79.2–99.3%), a reduction of the number of ears (2.4–82.7%), and a reduced plant height (40.1–75.3%) in response to artificial WDV infection (Vacke and Cibulka, 2001). A similar strong influence of WDV infection was observed for the majority of accessions in our panel of 500 wheat accessions, however, a broader range varying from 3.6 to 100% plant height reduction, 0–100% ear number reduction, and 23.2–100% yield reduction was detected.

Basically, domesticated wheat did not show generally higher infection rates than wild relatives in our panel. Another study showed that domesticated wheat did not always show stronger symptoms but the response to WDV infection on growth traits and leaf chlorosis was very variable for both wild and domesticated wheat species (Nygren et al., 2015). This observation suggests that the genetic bottleneck during evolution and domestication of wheat did not necessarily result in higher WDV susceptibility and might be compensated by new variation obtained through the hybridization of ancestors.

Initially, only two Hungarian winter wheat cultivars, “Mv Dalma” and “Mv Vekni,” were described to be partially resistant (Benkovics et al., 2010). In vector transmission assays, they showed 50% infected plants whereas the susceptible varieties “Mv Emese” and “Mv Regiment” had a significantly higher infection rate of 100%. Four weeks after the infection, milder virus symptoms were detected and a 100–10,000 times lower virus titer was measured for the partially resistant varieties. Our screening confirmed the lower average infection rates of “Mv Dalma” (34.5%) and “Mv Vekni” (21.5%) and weaker symptom expression compared to the susceptible varieties. Further, we could also identify 19 additional sources of WDV resistance with a lower infection rate than “Mv Vekni” (Supplementary Table S2) including di-, tetra-, and hexaploid gene bank accessions giving hint that natural sources of WDV resistance are present in the wheat gene pool. For breeding purposes, the registered Russian winter wheat variety “Fisht” with 5.7% infected plants on average over three test seasons in the gauze houses may be of special interest.

### Identified Quantitative Trait Loci for *Wheat Dwarf Virus* Resistance in the Association Panel of Hexaploid Wheat

As expected, the average LD of the association panel (2.97 cM) is lower than those determined in other GWAS studies using advanced wheat cultivars (Breseghello and Sorrells, 2006; Lehnert et al., 2017). The lower LD distance might reflect the higher genetic diversity present in gene bank accessions in contrast to modern cultivars. The number of resistant genotypes within our panel was rather low and therefore the usual minor allele frequency of MAF > 5% (Alqudah et al., 2020) was reduced to MAF > 3% in order to take rare alleles into account.

A total of thirty-five putative QTL have been identified (FDR, α < 0.05) for partial WDV resistance located on 11 chromosomes for the traits relPH, relYield, and relTKW. For the traits E and IR, significant MTAs according to the FDR correction could not be detected in the GWAS panel, however, significant effects for E were detected in the biparental populations. This might be reasoned in the different genotypes contributing to WDV resistance in the association panel and the biparental populations.

For the trait E, the QTL_PH_1B1-1B4, QTL_PH_4A3, and QTL_Yield_1B1-1B4 are significant. Therefore, the effect can possibly be attributed to pyramidization. According to our knowledge, QTL for WDV resistance have not been described in wheat so far. But several GWAS studies successfully described the identification of QTL for the resistance of other vector-transmitted virus diseases in cereals. For example, six QTL for resistance to the aphid-transmitted BYDV in spring oat lines were detected by using an oat SNP array (Foresman et al., 2016). A total of eight QTL (FDR, α < 0.05) for BYDV resistance investigating virus extinction and infection rate in maize have been identified by association analysis (Horn et al., 2014). New sources of resistance to BYDV have been found in a panel of 335 wheat gene bank accessions (landraces) from different locations using the 90k iSelect Illumina chip and four novel QTL were detected by GWAS (Choudhury et al., 2019).

Regarding WDV, resistance was detected in the barley cultivar “Post” on chromosome 2H by biparental QTL mapping (Habekuß et al., 2009). In the DH population, “Post” x “Vixen,” used in this study, a continuous Gaussian distribution for plant height and degree of attack was observed indicating a polygenic inheritance of WDV resistance in barley. Similarly, our GWAS results indicate that the partial resistance in wheat is caused by several QTLs (FDR, α < 0.05) distributed on chromosomes 1B, 1D, 2B, 3A, 3B, 4A, 4B, 5A, 6A, 7A, and 7B. Previous studies detected that resistances to different viruses in wheat were often located on the D genome, e.g., SBWMV resistance was identified on chromosomes 4D and 5D by association analysis of 205 wheat accessions (Zhang et al., 2011; Liu et al., 2014). One resistance encoding allele on chromosome 5D was traced back to *Ae. tauschii.* In another GWAS, a highly significant MTA (LOD = 31) for resistance to *Wheat spindle streak mosaic virus* (WSSMV) was detected on chromosome 2D besides the additional MTAs on chromosomes 2A, 2B, 3B, 5B, 5D, 7A, and 7B (Hourcade et al., 2019). However, in our study, the majority of significant MTAs for WDV resistance were detected on chromosome 1B. In particular, 6 validated QTL explaining > 10% of the phenotypic variance and a LOD between 5.0 and 8.7 were identified, especially the highly significant yield-related QTL WDV_Yield_1B1 and WDV_Yield_1B3 explaining 18.3% of the phenotypic variance might be considered for further development of molecular markers in resistance breeding.

### Quantitative Trait Loci Annotation Reveals Genes for Stress Response

The annotation of the genes located in respective QTL regions identified genes known to be involved in various stress responses in plants (Supplementary Table S4). In this study, genes involved in DNA templated regulation of transcription, messenger RNA (mRNA) splicing *via* spliceosome, gene silencing by RNA, and kinase activity were detected.

The QTLs WDV_PH_5A4, WDV_Yield_1B3, and WDV_TKW_5A1 include six genes which encode DNA templated regulation of transcription. These may act as viral defense modulators with respect to the host-dependent DNA replication cycle of WDV (Gutierrez et al., 2004).

The gene TraesCS2B01G138700 encodes gene silencing by RNA and is located in the region of the QTL WDV_Yield_2B1 on chromosome 2B. Previous studies have shown that geminiviruses can induce post-transcriptional gene silencing (PTGS) (Chellappan et al., 2004; Li et al., 2017).

Additionally, the QTL intervals of WDV_PH_5A4, WDV_Yield_1B3, WDV_Yield_2B1, and WDV_Yield_2B2 contain high confidence genes, which encode several protein kinase domains. Studies showed that kinases are involved in plant resistance to geminiviruses. For instance, SNF1-related kinases phosphorylate the viral ßC1 pathogenesis protein. Phosphorylation negatively affects the RNA silencing suppressor function of ßC1 or it is marked for degradation in the 26s proteasome. As a result, reduced/delayed viral infection is observed (Shen et al., 2012).

Our results suggest that further resistance genes might also be involved in geminivirus defense, which requires further investigation.

### Verification of Quantitative Trait Loci for *Wheat Dwarf Virus* Resistance in Four Populations

We used four different populations with the cv. “Fisht” as the resistant parent to verify the identified QTL. A total of 25 QTLs were tested explaining between 7.4 and 18.3% of the phenotypic variance. Eleven QTLs were filtered out due to MAF and heterozygosity. Out of the remaining 14 QTLs, six are major QTLs i. e. explaining more than > 10% of the phenotypic variance (Collard et al., 2005). Segregation analysis confirmed that two markers had significant effects for the relYield, 13 for the relTKW, and 10 for the relative virus titer (Table 4 and Supplementary Table S7). In this study, the QTLs on chromosome 1B consistently showed highly significant effects in the four populations as well as the markers on chromosome 3A and 5A. For further validation of these QTLs, it has been planned to create a linkage map based on the populations and to conduct biparental QTL analysis.

In other studies, it was already successfully demonstrated that QTL, which have been detected in association genetic studies, are relevant for breeding purposes and can be verified in biparental populations. For example, MTAs for important agronomic traits were confirmed in DH populations derived from barley lines having positive alleles for these traits in the same way as in our study (Lüders et al., 2016), or QTL for mildew resistance previously identified by GWAS were validated in wheat (Li et al., 2019).

The effects of the markers on the characteristics were not identical between simple and multiple regression models (Supplementary Table S8). These differences may be caused by relationships between the markers. The measures of determination between actual and predicted values showed that the models based on individual population data sets had higher predictive accuracy than the model based on Pop_total (Supplementary Figure S4). This suggests that the model should be built based upon individual biparental populations because the number and effects of QTL, which segregate in a biparental population, vary between the crosses. Three of the DH populations have “Fisht” as the maternal parent. Therefore, the differences observed between the populations in the regression models may be due to differences in the genetic background of the paternal parents.

Not all identified markers showed positive effects in each of the four populations. Nevertheless, higher prediction accuracy was achieved when all markers were used, as opposed to using only the significant markers alone. Therefore, in terms of practical breeding, building a regression model using all QTL identified in this study by GWAS is an attractive approach to increase selection efficiency for WDV resistance.

### Marker-Assisted Selection for *Wheat Dwarf Virus*

The usefulness of these QTLs in improving WDV resistance through breeding depends on their robustness, i.e., their ability to predict effects in a range of genetic backgrounds. For breeding, the phenotypic variance explained by QTL is important (Collard et al., 2005). A QTL should explain more than 10% of the phenotypic variance for application in marker-assisted selection (Miedaner and Korzun, 2012). It has been shown that large QTL (>10% phen. var. expl.) associated with resistance remained stable across different environments (Li et al., 2001; Lindhout, 2002; Pilet-Nayel et al., 2002). In this study, six QTLs explained a phenotypic variance > 10%. The pyramidization of these QTLs thus represents an interesting approach to increase the resistance level to WDV (Parlevliet, 2002; Palloix et al., 2009; Brown et al., 2015). This has already been shown for BYDV in barley (Riedel et al., 2011).

To access the detected QTL in applied wheat breeding, the verified array-based markers can be used to develop PCR-based markers that can be used for marker-assisted selection. For example, kompetitive allele-specific PCR (KASP) markers may be developed from flanking SNP marker sequences. The efficiency of this approach has been shown in hexaploid wheat (Ayalew et al., 2019; Karlstedt, 2020). It provides a reliable and cost-effective method for marker-assisted selection in wheat.

Although the cost of genotyping has decreased in recent years, access to array-based systems is still costly. This hinders the adoption of array-based systems in plant breeding programs. Due to polyploidy, difficulties arise in wheat in distinguishing between allelic and genomic SNPs (Allen et al., 2013). Most SNP resources originate from exonic sequences (Akhunov et al., 2010; Allen et al., 2011). These tend to have greater similarities in the A and B genomes than intronic sequences. For a more precise detection of SNPs, the development of genome-specific primers may be helpful.

## Conclusion

The screening for WDV resistance in wheat and wild relatives showed that differences in the susceptibility to WDV exist in the wheat gene pool. According to the results obtained, interesting *T. aestivum* sources of partial WDV resistance might be the gene bank accession PI 245511 and the Russian winter wheat cultivar “Fisht,” which exhibited low average infection rates of about 5% and relative yields compared to the healthy control of 77 and 55%, respectively. Through association genetic studies on hexaploid *Triticum* species, we were able to identify 35 QTLs for WDV resistance. The effect of 25 QTLs was confirmed in biparental populations. In particular, we identified 14 QTLs that were consistently associated with low losses in relative yield, relative thousand-grain weight, and low relative virus titer across different genetic backgrounds. The introduction of these QTL from “Fisht” into elite winter wheat cultivars through MAS may be a promising method to improve resistance to wheat dwarf disease in wheat.

## Data Availability Statement

The datasets presented in this study are deposited at OpenAgrar under https://doi.org/10.5073/20220203-155132.

## Author Contributions

AH and FO planned and designed the research. A-KP and BR conducted the experiments, data analysis, and wrote the manuscript. AH, TW, AS, KP, and FO contributed to the interpretation and discussion of results. All authors contributed to the article and approved the submitted version.

## Conflict of Interest

The authors declare that the research was conducted in the absence of any commercial or financial relationships that could be construed as a potential conflict of interest.

## Publisher’s Note

All claims expressed in this article are solely those of the authors and do not necessarily represent those of their affiliated organizations, or those of the publisher, the editors and the reviewers. Any product that may be evaluated in this article, or claim that may be made by its manufacturer, is not guaranteed or endorsed by the publisher.
