# Corrigendum: Identification and validation of Quantitative Trait Loci for *Wheat dwarf virus* resistance in wheat (*Triticum* spp.)

**DOI:** 10.3389/fpls.2022.1088938

**Published:** 2022-12-19

**Authors:** Anne-Kathrin Pfrieme, Britta Ruckwied, Antje Habekuß, Torsten Will, Andreas Stahl, Klaus Pillen, Frank Ordon

**Affiliations:** ^1^ Julius Kühn Institute (JKI) – Federal Research Centre for Cultivated Plants, Institute for Resistance Research and Stress Tolerance, Quedlinburg, Germany; ^2^ Institute for Agricultural and Nutritional Sciences, Plant Breeding, Martin-Luther-University of Halle-Wittenberg, Halle (Saale), Germany; ^3^ Julius Kühn Institute (JKI) – Federal Research Centre for Cultivated Plants, Quedlinburg, Germany

**Keywords:** *Wheat dwarf virus* (WDV), genome-wide association study (GWAS), wheat (*Triticum aestivum*), quantitative trait loci (QTL), resistance breeding, *Psammotettix alienus*

In the published article, there was an error in **Table 4**. **Table 4** represents the P values of Supplementary Table S7. In Table S7, false P-values and means were reported for the populations FixFa, FixS, FixRe, and RexFi. After their correction, the total number of significant differences decreased. However, there are no differences in the QTL that show significance. In conclusion, 14 of the 35 QTL that were identified by GWAS are shown to be significant. The overall statement remains unchanged. **Table 4** is mentioned in **Results** “Validation of Quantitative trait loci”. For this reason, an adjustment is also made in the text. The corrected **Table 4** and its caption appears below.

**Table 4:** Validations of the identified QTL using a t-Test, *, ** indicate significant differences between allele means at 5% and 1% level, respectively.

		Population total							Population each						
		total			FixFa			FixS			FixRe			RexFi	
		P			P			P			P			P	
QTL	refYield	relTKW	E	refYield	relTKW	E	relYield	relTKW	E	relYield	relTKW	E	refYield	relTKW	E
WDV_PH_1B1		0,015*	0,035	0,003*	0,003*							0,035			
WDV_PH_1B2		0,021	0,023*	0,006*	0,004							0,023			
WDV_PH_1B3		0,017*	0,027*	0,003*	0,003*							0,027*			
WDV_PH_1B4		0,021	0,035*	0,003*	0,003*							0,035			
WDV_PH_1B5		0,031		0,003*	0,003*							0.03"			
WDV_PH_2B1		0,008**													
WDV_PH_3A1															
WDV_PH_3B1	0,001**	0,015*				0,01"									
WDV_PH_3B2															
WDV_PH_3B3															
WDV_PH_4A3			0,006**												
WDV_PH_4B1															
WDV_PH_5A1															
WDV_PH_5A2															
WDV_PH_5A3															
WDV_PH_6A1			0,00001**			0,032*									
WDV_PH_7A1	0,02*														
WDV_PH_7A2															
WDV_PH_7A3															
WDV_Yield_1B1		0,021	0,023	0,01	0,008**							0,036			
WDV_Yield_1B2		0,017	0,027*	0,01	0,009**							0,031			
WDV_Yield_1B3		0,021	0,035	0,003**	0,007**							0,036			
WDV_Yield_1B4		0,026	0,027	0,01	0,008**	0,03"									
WDV_Yield_2B1															
WDV_Yield_2B2															

There were also errors in Supplementary Tables S7 and S8. In Table S7, false P-values and means were reported for the populations FixFa, FixS, FixRe, and RexFi. After their correction, the total number of significances has decreased. However, there are no differences in the number of QTL that show a significant difference. In the conclusion, 14 of the 35 GWAS QTL are shown to be significant. The statement remains unchanged.

In Supplementary Table S8, the determined values of the simple and multiple regression analysis are given. An error occurred in the simple regression analysis of FixS and FixRe (values of FixFa were accidently given in both columns). However, the correction only affects this table, as it is not described in the results section. The values for the multiple regression analysis are correct.

The description of the validated QTL had to be changed because they referred to the error in **Table 4**. A correction has been made in **Results** “Validation of Quantitative trait loci”. This sentence previously stated:

“One QTL for relYield and ten for the extinction showed significant effects to varying degrees across all populations. The most significant effects were observed for TKW with 11 significant QTL (**Table 4** and Supplementary Table S7). Looking at the significances at the population level, most were observed for the FixRe population. In particular, four QTL for relPH and four QTL for relYield on chromosome 1B (WDV_PH_1B1, WDV_PH_1B2, WDV_PH_1B3, WDV_PH_1B4, WDV_Yield_1B1, WDV_Yield_1B2, WDV_Yield_1B3, WDV_Yield_1B4) showed highly significant and consistent effects in all populations. The effects of WDV_PH_7A1 for the trait relYield and those of WDV_Yield_1B2- 1B4 were only validated in two populations. An effect of WDV_PH_4A3, WDV_PH_6A1, WDV_Yield_1B1, WDV_Yield_1B3 and WDV_Yield_1B4 was only confirmed in the FixRe population.”

The corrected sentence appears below:

“Two QTL for relYield and ten for the extinction value (relative virus titer) showed significant effects to varying degrees across all populations. The most significant effects were observed for TKW with 11 significant QTL (**Table 4** and Supplementary Table S7). Looking at significant differences at the population level, most were observed for the FixFa population. In particular, one QTL for relYield on chromosome 1B (WDV_Yield_1B1) showed highly significant and consistent effects in all populations. The contributions of WDV_PH_1B1-1B5 and WDV_Yield_1B1-WDV_Yield_1B4 were significant in three populations. The effects of WDV_PH_7A1 were only validated in two populations. An effect of WDV_PH_3B1 and WDV_PH_6A1 was only confirmed in the FixFa population.”

The authors apologize for these errors and state that this does not change the scientific conclusions of the article in any way. The original article has been updated.

